# From filters to fairness: understanding colourism through TikTok aesthetics and trends

**DOI:** 10.3389/fsoc.2026.1731751

**Published:** 2026-08-28

**Authors:** Monica Ronniza Majumdar

**Affiliations:** Queen Mary University of London, London, United Kingdom

**Keywords:** algorithm, colourism, consumer behavior, digital studies, platform capitalism, social media, South Asian identities, South Asian women

## Abstract

This study investigates how British South Asian women engage with and interpret skincare and beauty content on TikTok, with particular attention to how the platform reproduces, normalizes, and commodifies colourism through its algorithmic and influencer-driven structures. The study is qualitative in design centring participants' own accounts of how TikTok's beauty culture shapes their self-perception, identity, and consumption practices. Data were collected from 15 participants via an online survey of open-ended qualitative questions, supplemented by a small number of descriptive Likert-scale items used solely as contextual framing. Participants' open-ended narratives form the exclusive analytical basis of the study and were analyzed using reflexive thematic analysis. The findings reveal that TikTok operates as both a site of creative self-expression and a mechanism that participants experienced as reinforcing colonial hierarchies of skin tone through subtle and overt colourist discourses. Participants reported that repeated exposure to brightening trends and influencer marketing heightened self-comparison and internalized beauty pressures. However, participants also demonstrated moments of resistance and critical reflection, challenging these ideals and reclaiming pride in their natural complexions. The tension between internalization and resistance emerges as the study's central analytical contribution: these positions coexist within individuals rather than representing opposing responses, revealing the structural ambiguity of agency within platform capitalism. Overall, the study concludes that TikTok's algorithmically personalized content simultaneously reinforces and exposes colourism, shaping how British South Asian women understand and negotiate identity amid digital empowerment and structural inequality.

## 1 Introduction

Over the past decade, social media platforms have become central infrastructures through which beauty norms, identity formation, and consumer practices are produced and circulated (Matthew, 2024). TikTok positions itself as a departure from the aspirational perfection of platforms like Instagram. Its emphasis on user-generated content and a highly personalized “For You” feed constructs a sense of intimacy and spontaneity between creators and audiences. This performative realism, where creators appear unfiltered, casual, and emotionally candid, renders the platform an influential arena for shaping perceptions of beauty, identity, and self-care (Taylor, 2025). It is this combination of apparent authenticity and structural commercial logic that makes TikTok a particularly consequential space for understanding how colourist ideals are reproduced and negotiated among British South Asian women.

Within TikTok's rapidly evolving beauty culture, skincare discourses centered on brightening, whitening, and glow have gained significant traction (Kang et al., 2024). Although framed through the language of wellness, self-care, and personal improvement, these terms echo longer histories of colourism that privilege lighter skin tones as markers of desirability, success, and social value. For British South Asian women, shaped by postcolonial legacies, diasporic identities, and racialised beauty hierarchies, TikTok represents a contradictory space. It offers visibility and opportunities for self-representation while also reproducing subtle forms of exclusion. Participants in this study describe experiencing algorithmic amplification and aesthetic standardization as forces that shape which bodies and skin tones are made visible; perceptions that, regardless of whether they can be verified against platform mechanics, carry real consequences for self-image and belonging. Such dynamics are best understood in relation to the historical formation of colourism within South Asian communities (Punathambekar et al., 2022).

Colourism has long structured social relations and gendered beauty norms within South Asian contexts, privileging lighter skin tones (Dixon and Telles, 2017). Shaped by colonial legacies, caste hierarchies, and global white supremacy, fairness has been linked to femininity, respectability, and social mobility (Glenn, 2008; Hunter, 2007; Nagar, 2018). Under British colonial rule, lighter skin became associated with power and proximity to whiteness, while darker skin was devalued, entrenching hierarchies that persist today (Hall, 1990). The persistence of these hierarchies is not incidental; it reflects their deep embedding in social institutions, family norms, and cultural imaginaries that digital platforms have amplified rather than dissolved.

In contemporary contexts, colourism operates through everyday practices such as family expectations, beauty advertising, and the promotion of brightening products (Dixon and Telles, 2017; Hunter, 2007; Ellis and Destine, 2023). It is sustained not only through overt discrimination but also through routine pressures to manage appearance. As such, colourism remains an adaptive system shaping women's self-perception across both offline and digital spaces.

Against this backdrop, this paper examines how British South Asian women perceive and interpret skincare content on TikTok, focusing on how repeated exposure to brightening and glow-orientated skincare discourses shape user perceptions and practices within colourist beauty narratives. The study focuses on terminology such as brightening, glow, and radiance because existing research has shown that such terms operate as euphemistic substitutes for skin lightening (Glenn, 2008; Parameswaran and Cardoza, 2009). Importantly, participants' accounts reflect how they experience and interpret the platform's content; the distinction between perceived algorithmic behavior and demonstrable platform mechanics is maintained throughout.

This paper makes three key contributions. First, it extends research on colourism by situating skin-tone hierarchies within contemporary algorithmic media environments, showing how historically rooted ideals of lightness are sustained and experienced through platform infrastructures. Second, it contributes to digital media studies by demonstrating how TikTok shapes racialised perceptions of desirability, authenticity, and consumer identity as narrated by British South Asian women themselves. Finally, by centring these women's qualitative accounts, it advances feminist and diasporic media research, illuminating how users navigate, interpret, and at times resist colourist discourse within participatory yet commercialized digital spaces.

## 2 Literature review

### 2.1 The role of TikTok in shaping contemporary beauty and skincare culture

Social media platforms have become integral to everyday life and cultural production, reshaping how beauty, identity, and self-presentation are negotiated. Researchers increasingly recognize that these platforms are not merely communication tools but digital ecosystems that produce and circulate aesthetic values (Abidin, 2023; Duffy, 2017). Within this digital ecology, TikTok has become one of the most influential visual spaces (Kułaga, 2024), particularly among younger demographics. Kushwaha (2024) observes that social networking sites offer new possibilities for self-expression and the re-articulation of femininity, allowing women to experiment with self-presentation beyond the constraints of traditional media. There is genuine evidence for this: studies of TikTok's non-relational social model have found that it enables creators from marginalized communities to build audiences and visibility without the established follower networks that previously gatekept mainstream media exposure (Sujon and Ntalla, 2025; Blackburn and Hogg, 2024). This democratic affordance is real and should not be dismissed. However, as Banet-Weiser (2018) argues, social media's politics of authenticity, the logic that promises empowerment and individuality, remains entangled with consumer capitalism in ways that complicate straightforward optimism about representation.

In the UK, TikTok's impact on the beauty and skincare industry is striking. The platform now boasts 30 million active users, and TikTok shop has become the fourth-largest beauty retailer in the country, with over 498 billion beauty-related views (CEW UK, 2025; Beauty Matter, 2025). Its architecture, based on short-form, vertically scrolling videos and a hyper-personalized “For You” page, creates an immersive environment where trends emerge and decay at remarkable speed. Within this environment, visibility becomes contingent on continuous self-presentation, producing what Duffy (2017) terms “aspirational labor”: creators, particularly in the beauty sector, engage in ongoing affective and aesthetic work to remain visible and maximize opportunities for monetisation.

The platform's algorithm personalizes the For You page to curate an ever-evolving stream of skincare routines, product recommendations, and transformation videos (Ebrahim and Tanner, 2021). As Seekis and Lawrence (2023) explain, its recommendation system learns continuously from user behavior to surface content most likely to sustain engagement, meaning that videos aligning with dominant beauty norms are, as participants consistently described, more likely to be repeatedly surfaced in their feeds. Beauty ideals such as glass skin, a Korean-beauty ideal associated with an even, poreless, and luminous complexion, alongside glow, and brightening thereby circulate at an accelerated pace, collapsing the boundaries between authenticity, aspiration, and advertising. Despite TikTok's positioning as a platform of authenticity, its algorithmic and aesthetic infrastructures frequently produce the opposite effect: Felimban (2025) argues that visual-first platforms project an idealized world in which highly edited appearances are presented as both authentic and desirable. For British South Asian women, whose skin tones and features diverge from the platform's dominant aesthetic, this gap between performed authenticity and lived appearance is not merely cosmetic; it is the space in which colourist norms take hold. Colourist beauty ideals do not need to be explicitly promoted to be effectively transmitted; repetition alone does the ideological work.

Simultaneously, TikTok operates as an emblem of platform capitalism (Srnicek, 2017), where user participation, emotional labor, and self-representation are transformed into sources of profit. Creators in the beauty and skincare niches function as both cultural intermediaries and economic actors, commodifying relatability, and authenticity as forms of brand value (Duffy, 2017; Banet-Weiser, 2018). It is vital to acknowledge that platform capitalism as a framework has attracted criticism for its tendency toward structural determinism; for presenting users primarily as subjects of commercial extraction rather than as agents capable of exploiting platform affordances for their own ends (Postigo, 2016; van Dijck et al., 2018). Critics argue that the framework risks underestimating the extent to which users, including those from marginalized communities, actively shape platform cultures rather than passively receiving them. This critique has merit, and the present study is attentive to it: the qualitative data consistently shows participants who are not only subject to commercial logic, but who analyse, critique, and at times subvert it. The framework of platform capitalism is retained here not to deny this agency, but to insist that such agency is exercised within structural conditions that the framework helps make visible. The speed of these interactions also fosters cycles of comparison, aspiration, and consumption, consistent with social comparison theory (Festinger, 1954; Vogel et al., 2014).

Overall, the literature presents TikTok as a hybrid socio-technical marketplace that merges the affective appeal of authenticity with the commercial imperatives of digital capitalism. While this globalized visibility offers space for new forms of representation, it also reproduces subtle hierarchies of desirability, particularly around skin tone and complexion. The central tension this section has established is that TikTok's democratic promise and its colourist effects are not in contradiction; they are produced by the same mechanisms. Authenticity, relatability, and community are the platform's cultural offer; engagement optimisation and commercial scale are its structural logic.

### 2.2 Colourism in the digital space

Colourism, broadly defined as the privileging of lighter skin tones within and across racial and ethnic groups, has long functioned as a mechanism through which social hierarchies of beauty, class, and desirability are maintained (Phoenix and Craddock, 2025). Its origins are deeply entangled with the colonial and imperial histories that equated whiteness with modernity, purity, and progress, while marking darker skin as inferior. Hunter (2007) and Glenn (2008) argue that skin-tone hierarchies persist across societies and diasporas, shaping employment prospects, marriage markets, and cultural ideas of femininity. Within South Asian contexts, fairness remains a signifier of privilege, upheld through centuries of caste stratification and colonial mimicry.

Hall (1990) and Dyer (1997) argued that visual culture is central to the construction of race; advertising, camera and cinema have historically illuminated whiteness as the normative standard of beauty and humanity. Hall's (1990) emphasis on representation as an active meaning-making process highlights how visual media do not merely reflect racial hierarchies but participate in their reproduction; a framework that remains crucial for analysis of contemporary digital beauty cultures. In beauty advertising, for example, lighter skin has long symbolized success, cleanliness, and aspiration, while darker tones are coded as undesirable or in need of correction (Parameswaran and Cardoza, 2009). This visual grammar of correction, the idea that darker skin is a problem to be solved, is what positions media visibility as both inclusion and control, granting recognition to bodies that conform to the dominant aesthetic while marginalizing those that do not.

In the digital era, these hierarchies persist through perceived algorithmic amplification and influencer visibility (Taylor, 2025). Research by Love et al. (2025) found that users reported being less likely to click on videos featuring dark-skinned influencers. This finding relates to user behavior and audience preference rather than the algorithmic behavior of TikTok's recommendation system directly. It is important to acknowledge a more optimistic strand of scholarship here: researchers have found that TikTok's non-relational algorithm, by surfacing content based on engagement rather than existing social networks, has in certain contexts amplified the visibility of creators from underrepresented communities who would have struggled to build audiences on follower-dependent platforms (Bhandari and Bimo, 2022; Blackburn and Hogg, 2024). This optimistic strand of the literature is important and should not be dismissed; the question this study pursues is not whether visibility is possible on TikTok but under what conditions it is distributed and whose skin tones are centered when it is.

Beauty communities organized around South Asian, Black, and dark-skinned aesthetics have found genuine audiences on TikTok, and hashtags such as #BrownSkinGirl and #DesiGlow have attracted substantial engagement. This evidence of broadened representation is real and should not be discounted. However, as the insights from this study show, the existence of representational space does not guarantee its equitable distribution or freedom from colourist hierarchies: participants encountered both moments of genuine visibility and the persistent dominance of brightened, lighter-skin toned aesthetics in their feeds. The two experiences coexist, and it is that coexistence, rather than either harm or empowerment taken alone, that the study is positioned to investigate.

Research on digital beauty filters further underscores how technology operationalises colourist ideals. Xu et al. (2023) document that TikTok hosts thousands of filters designed to modify facial features, including smoothing skin texture and lightening complexion. A growing body of empirical research links frequent engagement with such filters to increased body dissatisfaction and appearance-related anxiety (Schröder and Behm-Morawitz, 2025; Wang, 2024; Caravelli, 2025). Through this lens, TikTok's participatory culture is both empowering and disciplining: users can represent themselves, yet they often do so within templates of beauty that participants experience as algorithmically rewarded, and that valorise lighter skin tones.

For diasporic communities, particularly British South Asian women, the encounter with these digital aesthetics is complex. Living within a multicultural society that celebrates diversity yet continues to privilege Eurocentric standards, these women navigate intersecting discourses of race, gender, and class. Scholars of the South Asian diaspora note that colourism persists as a transnational ideology transmitted through family expectations, popular media, and beauty commerce (Parameswaran and Cardoza, 2009; Saha, 2021). Yet, the relationship between South Asian diasporic identity and colourist beauty norms is neither static nor uniform. Phoenix and Craddock (2025) find that younger British South Asian women are increasingly contesting intergenerational colourism rather than simply inheriting it, and Punathambekar et al. (2022) show that diasporic media cultures can generate new forms of self-representation that challenge rather than replicate dominant beauty ideals.

Colourism within South Asian communities is better understood as a contested ideological terrain than as a monolithic value system, which is precisely what makes its reproduction through digital platforms analytically interesting. On TikTok, hashtags like #GlowUp, #BrighteningRoutine, and #RadianceSkin circulate widely, often repackaging colourist language in wellness and self-care vernacular (Kang et al., 2024), reproducing hierarchies even among users who would explicitly reject them if named directly.

Digital spaces are not solely sites where colourist hierarchies are reproduced; they also function as arenas for counter-narratives, reflexive engagement, and forms of resistance (Tate, 2018; Jackson et al., 2020). Movements such as #BrownSkinGirl and #DarkIsBeautiful illustrate how users actively contest internalized colourism through community storytelling and digital activism. Drawing on Tate's (2018) work on the governmentality of beauty shame, such engagements can be understood as conscious interventions through which users negotiate, reframe, and at times refuse dominant beauty discourses. At the same time, as Banet-Weiser (2018) and Gill and Orgad (2021) put forward, acts of resistance are often absorbed and circulated as forms of marketable authenticity, a tension explored further in the discussion.

Overall, the literature on colourism in the digital space reveals a paradox: digital platforms promise visibility, voice, and empowerment, but they remain structured by historical hierarchies of lightness that are now encoded in algorithmic design and influencer economies. For British South Asian women, navigating TikTok's skincare culture means negotiating between representation and regulation, authenticity, and aspiration.

### 2.3 TikTok and colourism: intersecting discourses of beauty and technology

Empirical research highlights persistent racial imbalances within TikTok's beauty culture, consistent with Srnicek's (2017) and Noble's (2018) observation that platforms structured around engagement optimisation tend to amplify normative beauty content, though the specific relationship between engagement signals and algorithmic outputs is not directly observable to users and this study does not claim to verify it. Harriger et al. (2023), in their analysis of 342 videos tagged under body positivity, found that over 93% reflected Western beauty ideals, with nearly 69% featuring white women and only 2.3% featuring Asian representation. Harriger et al. (2023) note, however, that TikTok's body positivity content showed greater diversity in terms of body size than comparable content on Instagram, suggesting that the platform's algorithmic architecture can, in some dimensions, facilitate broader representation, even where racial diversity remains severely underrepresented. This distinction matters for this study: TikTok's democratic affordances are not uniformly absent, but they appear to operate unevenly across axes of identity, with racial and skin-tone diversity lagging behind representational progress. This pattern can be understood through the lens of intersectional invisibility, whereby individuals who occupy multiple marginalized identities are rendered less visible within spaces ostensibly designed to promote inclusion (Purdie-Vaughns and Eibach, 2008), and it underscores the particular importance of foregrounding British South Asian women's experiences.

TikTok received significant criticism for promoting unrealistic beauty ideals through the release of its Bold Glamor filter (Allyn, 2023). The effect is hyper-realistic and nearly undetectable, making it increasingly difficult for users to distinguish between filtered and unfiltered appearances. The Bold Glamor controversy also exposes the contradictions of TikTok's brand identity: an app celebrated for authenticity that simultaneously promotes aesthetic perfection through computational enhancement. Studies have shown that face-altering filters encourage users to compare their offline appearance with digitally enhanced versions of themselves, intensifying self-surveillance (McLean et al., 2015; Veldhuis et al., 2020; Hermans et al., 2022; Schröder and Behm-Morawitz, 2025). Where filters encode lighter, smoother skin as the enhanced version of the self, the self-surveillance they intensify is not aesthetically neutral; it is colourist in its terms of reference.

Social comparison theory (Festinger, 1954; Vogel et al., 2014) provides a useful framework for understanding how users evaluate themselves in relation to others within TikTok's visual economy. It is worth acknowledging that social comparison research has been critiqued for overemphasizing upward, negative comparisons while underweighting the role of lateral and downward comparison, social support, and community solidarity in shaping self-evaluation (Vogel et al., 2014; Tylka et al., 2023). Research on body-positive communities specifically has found that exposure to diverse representation can produce positive self-evaluation effects under certain conditions (Cohen et al., 2019). The framework is retained here not as a complete account of how social comparison operates on TikTok but because it captures the specific dynamic, continuous upward comparison facilitated by an algorithmically curated feed of aspirational content, that the participants in this study consistently describe. Empirical support for this comes from Seekis and Kennedy (2023), who found that exposure to beauty content on TikTok was associated with increased appearance shame and upward comparison processes among young women, with self-compassion serving only a partial moderating role. These processes must also be situated within longer histories of racialised aspiration, through which the language of brightening, glow, and radiance rearticulates established colourist ideals as wellness and self-care. Even counter-narratives intended to challenge idealization may inadvertently sustain hierarchies by positioning whiteness as the baseline from which authenticity is measured (Harriger et al., 2023).

Overall, the literature establishes TikTok as a space where beauty ideals, colourism, and digital visibility intersect in ways that carry real consequences for racialised users, but it has been more effective at documenting structural patterns than at capturing how individual women experience, interpret, and contest these dynamics in practice. This study addresses that gap by drawing on qualitative narratives to explore the depth and texture of these experiences among British South Asian women. Descriptive survey items are included to provide contextual orientation but do not constitute a parallel strand of analysis.

Accordingly, the study is guided by three key research objectives:

- To investigate how engagement with TikTok content shapes self-perceptions and aspirational beauty ideals among British South Asian women.
- To explore how British South Asian women experience and interpret TikTok skincare content using terms such as brightening, whitening, and glow, and how this language shapes their perceptions of ideal skin tone.
- To analyse whether and how TikTok's algorithmic and creator structures, as perceived by users, commodify colourist skincare narratives and influence British South Asian women's consumption patterns and perceptions of brightening products.

## 3 Materials and methods

This study is qualitative in design, employing in-depth open-ended survey responses to explore how British South Asian women engage with and interpret skincare and beauty content on TikTok, with a focus on algorithmic visibility, influencer culture, and colourist discourse. Data were collected via an online survey that included a small number of descriptive Likert-scale components alongside the qualitative prompts. These descriptive items are not treated as a quantitative strand; they provide contextual framing for the qualitative analysis and are summarized as descriptive counts only. The analytical and theoretical contribution of the study rests entirely with the qualitative data.

The sample comprised 15 British South Asian women; participants ranged in age from 18 to 54, with the majority falling between 25 and 40. Identification with South Asian heritage was assessed through self-reported ethnic background selection in the demographic section of the survey. Participants represented a range of South Asian ethnic backgrounds, including Bangladeshi (*n* = 4), Indian (*n* = 4), Pakistani (*n* = 4), Sri Lankan (*n* = 2), and mixed South Asian heritage (*n* = 1). Professional backgrounds included working professionals (*n* = 9), students (*n* = 3), and homemakers (*n* = 3). Participants were primarily based in urban and suburban areas across the UK, including London (*n* = 8), Birmingham (*n* = 4), Oxford (*n* = 2), and Manchester (*n* = 1). All participants reported regular use of TikTok and engagement with skincare and beauty-related content.

Participants were recruited using a snowball sampling strategy initiated within British South Asian digital and community networks. This approach facilitates access to individuals with shared cultural and digital experiences while reflecting the informal, interconnected nature of online communities.

Data were collected through an online questionnaire comprising 15 questions. Quantitative questions measured participants' engagement with TikTok skincare content and related purchasing behaviors using a five-point Likert scale (1 = Never, 5 = Always), enabling a descriptive summary of engagement patterns within the group. Qualitative open-ended prompts were designed to capture participants' emotions, responses, perceived pressures, and lived experiences in relation to skincare content and representation on TikTok. Participants were encouraged to elaborate freely, and no word limits were imposed. The prompts were:

- In what ways, if any, do TikTok skincare trends or videos affect how you feel about your natural skin tone?
- Have you ever felt pressured to use certain skincare products or routines because of the way skin tones are portrayed on TikTok?
- How do conversations about skin tone on TikTok compare to those you experience in your everyday life (e.g., with friends, family, or community)?

### 3.1 Ethical considerations

Ethical clearance for this research was obtained from the Queen Mary University of London Research Ethics Committee. All participants provided informed consent before participating and were reminded of their right to withdraw from the study at any time without consequence. The survey was hosted on a secure, GDPR-compliant platform, with all responses encrypted and stored securely. To protect participant anonymity, pseudonyms have been used in place of participant identifiers throughout this paper. No financial incentives were offered. Following data collection, responses were reviewed for coherence, relevance, and indicators of automated or non-genuine participation; no evidence of bot-generated or fraudulent responses was identified.

### 3.2 Data analysis

The Likert-scale items are summarized as raw counts within the findings section to provide contextual framing, for instance, establishing that the majority of participants in this group reported regular exposure to brightening terminology before the qualitative analysis explores how that exposure was experienced. These counts are not analyzed statistically, do not carry inferential weight, and are not treated as a separate strand of findings. They are presented solely to orient the reader to the scale and consistency of participants' engagement with the content under study.

Qualitative data were analyzed using reflexive thematic analysis, following Braun and Clarke (2021) approach. The analytical process moved through six phases: familiarization with the data through repeated reading; generation of initial codes applied across all responses; searching for themes by grouping codes into candidate patterns; reviewing and refining themes against the full dataset to ensure each was internally coherent and sufficiently distinct; defining and naming themes by articulating the central organizing concept of each; and producing the final analysis by selecting representative quotations and developing the theoretical argument. Codes were generated inductively, without imposing a predetermined framework, capturing recurring patterns relating to colourism, algorithmic visibility, influencer culture, self-perception, and resistance. Themes were not treated as fixed containers but as active interpretive constructs, consistent with Braun and Clarke (2021) insistence that reflexive thematic analysis foregrounds the researcher's role in meaning-making rather than treating themes as residing transparently in the data. Particular attention was paid to affective language, metaphor, and evaluative statements, allowing the analysis to capture both emotional responses and culturally situated interpretations of TikTok skincare content. Quotations are presented throughout the findings to support transparency and allow readers to evaluate the relationship between data and interpretation. An audit trail was maintained throughout, documenting coding decisions, theme revisions, and the rationale for interpretive choices, and all themes were reviewed against the full dataset before finalization to ensure internal coherence and minimize selective or anecdotal interpretation.

### 3.3 Researcher positionality

As a British South Asian woman, positionality informed both participant recruitment and data analysis in this study. Recruitment through community and digital networks was facilitated by shared cultural familiarity, which supported participant trust and openness when discussing sensitive topics such as colourism, beauty norms, and racialised self-perception. This shared positioning is a methodological strength in terms of access and depth of disclosure, but it also carries analytical risks: the researcher may unconsciously recognize and foreground experiences that mirror her own while underweighting those that diverge. To manage this, reflexive memo-writing was undertaken at each stage of the analytical process, explicitly documenting where the researcher's own experiences of colourism and digital beauty culture were activated by the data and where interpretive decisions were made. During coding, memos were used to distinguish between recognizing a pattern across participants' accounts and projecting a pattern onto them. The final themes reflected the range of participants' experiences rather than the most resonant subset. This process does not eliminate positionality as an influence on interpretation; reflexive thematic analysis does not claim to do so. It makes that influence visible and accountable, consistent with Braun and Clarke (2021) understanding of reflexivity as an active analytic practice rather than a declaration of neutrality.

## 4 Findings

The survey included five Likert-scale prompts alongside the open-ended qualitative questions. As background context: the large majority of participants reported frequent engagement with skincare-related TikTok content; almost all regularly encountered brightening, whitening, or glow terminology; most had purchased or considered purchasing brightening products following TikTok exposure; and all 15 reported at least occasional exposure to skin-altering filters. These observations establish that the experiences participants describe in their qualitative accounts were broadly shared within this group rather than exceptional. No analytical claims are drawn from them. The study's contribution rests entirely with the qualitative analysis that follows.

### 4.1 Performative self-presentation and the construction of digital beauty

Across participants' accounts, a recurring tension emerged around how beauty and authenticity are performed on TikTok. Rather than being a space of unfiltered self-expression, participants described a digital environment where self-presentation is shaped by algorithmic norms, influencer aesthetics, and subtle hierarchies of visibility. What participants describe is not simply exposure to beauty ideals but the felt pressure to perform those ideals, to align their online selves with dominant standards of glow, radiance, and brightness in order to belong. This transforms authenticity from a quality of self-expression into a form of aesthetic labor: looking natural, on TikTok, still requires work.

Anisa and Ruksana were asked how TikTok skincare content affects their relationship with their natural skin tone. Their responses reveal accounts that existing theories of colourism in advertising (Glenn, 2008; Parameswaran and Cardoza, 2009) cannot fully anticipate: that the colourist message is not delivered by a brand but absorbed through the accumulated aesthetic of ordinary users who appear to share the same skin aspirations.

**Anisa:** “A lot of the content I see pushes this idea that good skin means having a bright, even, almost glowing complexion, which often translates to lighter-looking skin. Even when the videos don't say it directly, there's a subtle message that darker or textured skin needs to be fixed or brightened to look beautiful. Seeing so many creators using toners, serums, and brightening masks made me feel like I needed to do the same just to keep up.”

**Ruksana:** “TikTok skincare trends sometimes make me feel a little insecure about my natural skin tone. I see so many videos promoting products that promise to brighten or even out skin, and it often feels like lighter or flawless skin is being idealized. Even if that's not the creator's intention, it can make me question whether my natural tone is something that needs fixing. It's a mix of admiration and frustration. I love learning about skincare, but I don't love feeling like my natural skin isn't good enough.”

Glenn (2008) and Parameswaran and Cardoza (2009) document how fairness is coded through advertising language, but Anisa describes a mechanism that precedes language entirely. The message that darker skin “needs to be fixed” is embedded in the visual messaging of who appears on screen, in what context, and with what products, a form of aesthetic conditioning that operates below the threshold of conscious persuasion. Hall (1990) enables an understanding of why this conditioning is so difficult to resist: because it arrives without a sender. There is no advertisement to dismiss, no brand to distrust. There is only the cumulative weight of thousands of creators whose skin looks the same way, all apparently arrived at that look through the same products. The ideological work is distributed across a community, which makes it feel like consensus rather than instruction; and consensus, unlike advertising, does not invite the skepticism that might allow it to be refused.

Ruksana's account adds a dimension that Anisa's does not explicitly name: the affective cost of maintaining critical awareness inside a system designed to erode it. Tate's (2018) framework on the governmentality of beauty shame describes how colourist norms are internalized as self-regulation but does not account for the exhaustion of knowing the system is operating on you and being unable to stop it from working. Ruksana knows that the ideal she is being offered is not her skin; she tells herself her skin is “unique and beautiful;” and she still feels the anxiety. What her account reveals is that critical consciousness and affective vulnerability are not opposites; they coexist, sometimes within the same sentence. Tate's (2018) concept of the governmentality of beauty shame holds that colourist norms operate through internalized self-regulation that persists even when the subject recognizes the system producing it: critical consciousness does not dissolve the norm; it coexists with it. As participants consistently describe it, the For You Page does not pause when the user becomes aware of what it is doing. Awareness is not a condition of exit. The feed, as participants described it, continues, and with it the comparison.

Taken together, the accounts of Anisa and Ruksana illuminate a dynamic that social comparison theory (Festinger, 1954; Vogel et al., 2014) begins to explain but does not fully contain. Festinger's (1954) original formulation holds that individuals evaluate their own opinions and abilities by comparing themselves to others, with upward comparison, measuring oneself against those perceived as superior, generating the greatest self-evaluative pressure. Vogel et al. (2014) extend this to social media environments, finding that passive exposure to idealized content produces more negative self-evaluation than active engagement, precisely because the viewer has no conversational resource with which to contextualize or contest what they are seeing. Both dynamics are illustrated here: Anisa describes a process of passive, repeated exposure to a visual standard she did not seek out and cannot easily avoid, while Ruksana articulates the self-evaluative consequence, the felt sense that her skin is something that “needs fixing.” What the participants' accounts add that social comparison theory cannot supply, however, is the specific ideological content of the comparison target. Social comparison theory is a structural account of a psychological mechanism; it does not explain why the standard being compared against encodes a racialised hierarchy of skin tone rather than some other dimension of appearance. For that, the theory must be read alongside Glenn (2008) and Hall (1990): the comparison is upward not merely because the creators on Anisa and Ruksana's feeds have clearer or brighter skin, but because that brightness carries the accumulated cultural weight of colourism, rendering it not simply aspirational but ideologically loaded. TikTok's For You Page does not create the terms of comparison; as participants experience it, it delivers them at scale, personalized and without pause.

The performative logic does not stop at self-perception; it restructures behavior, transforming skincare from a private practice into a public performance evaluated by an invisible audience whose approval is mediated entirely by the platform's aesthetic economy.

**Kavita:** “It's wild, the platform has turned skincare into a competitive sport. People aren't even washing their faces anymore; they're performing it for likes. And if your skin isn't glowing like a glazed donut under a ring light, you're clearly doing something wrong. The message is clear: your skin is only beautiful when it looks nothing like your actual skin.”

**Zulaykha:** “TikTok makes me feel like I'm committing a crime if I don't have a 10-step skincare routine imported straight from Seoul and blessed by the algorithm itself. Every influencer's face is glowing like a freshly waxed iPhone screen, and I'm over here wondering if I should start exfoliating my soul too.”

**Kiran:** “As a South Asian girl, that pressure isn't new; it's something that's always been around in subtle ways. But TikTok amplifies it. When creators talk about brightening or glow-up routines, it can sound a lot like the same old colourism, just with trendier language and packaging. I've even caught myself wanting to buy products I never cared about before, just because everyone online made it seem like having glassy or luminous skin was the goal.”

Duffy (2017) theorizes aspirational labor as the work performed by creators, the ongoing effort of self-presentation required to remain visible and monetisable on platform economies. What Kavita shows is that this logic has migrated from producers to consumers: the audience is now also performing. Banet-Weiser (2018) provides an understanding of why this migration has occurred. Her argument that platform cultures collapse the distinction between lived experience and performance, transforming everyday practices into bids for social and commercial validation, applies precisely here: TikTok's architecture, the Like button, the view count, the comment, makes any act of self-care into a potential bid for social validation. The platform does not distinguish between content and life; it simply renders all life as potential content, and rewards those whose life performs most legibly within its aesthetic economy.

Zulaykha's account adds the experiential texture that Ionescu and Licu (2023) theorize: it names the specific affective experience of being governed by an aesthetic standard one did not choose and cannot locate. The algorithm she describes as “blessed” is ironic but precise; it is not a person, not a brand, not even a set of rules she can identify and reject. Ionescu and Licu (2023) argue that TikTok's algorithm, as experienced by users, does not merely reflect identity but actively shapes it, progressively narrowing the aesthetic window through which users understand themselves as acceptable. What Zulaykha adds to their framework is the phenomenology of that narrowing: the felt experience of a system that has learned her preferences and now uses them against her, one she can recognize and name, but cannot refuse, because the standard it imposes was built from her own prior engagement.

Kiran's account is the most theoretically significant of the three because it makes explicit what the others imply: that TikTok's colourist aesthetics are not new but are a new delivery mechanism for an old ideology. Glenn (2008) and Parameswaran and Cardoza (2009) document the long history of fairness commodification in South Asian beauty culture; Kiran confirms that this history is not merely continuous with the present but has been accelerated and personalized by it. What her account adds that Glenn (2008) and Parameswaran and Cardoza (2009) cannot supply is the subjective experience of that acceleration, of recognizing the same pressure she has always felt arriving through a different interface, with trendier language and a checkout link already attached. Srnicek (2017) explains why the new delivery mechanism is more effective than the old one: platform capitalism does not merely circulate existing cultural content, it optimizes it, amplifying the forms that generate the most engagement and embedding commercial infrastructure directly at the moment of aspiration. The pressure to brighten that Kiran's community has long transmitted has now arrived through a personalized feed, timed to the moment her prior viewing history has made her most receptive. The ideology is the same. The system delivering it has been engineered for conversion.

### 4.2 *Influencer* culture, commercialization, and the commodification of beauty

Srnicek (2017) argues that platform capitalism extracts value from user participation, transforming self-expression into a commodity. Duffy (2017) extends this through the concept of aspirational labor; the affective and aesthetic work creators perform to remain visible and monetisable. What neither framework fully accounts for is how this commercial logic operates at the moment of consumption: how colourist desire is not simply available to be purchased but is actively produced in users who did not previously hold it. The accounts below make this production process visible.

**Meera:** “At least in real life, people pretend it's about personal preference. On TikTok, they skip the pretending and go straight to monetising the insecurity. It's not a conversation anymore, it's capitalism with good lighting. It's so easy to buy things directly from the app. The products are cheap, always on sale, and promoted in such convincing ways that it's hard not to be tempted. There are almost no restrictions, so anyone can sell creams or serums that promise lighter or clearer skin, even if they might not be safe or effective.”

**Naina:** “Not from people I know, but from the silent judgment of TikTok's For You Page. Before you know it, you're halfway through a cart full of serums promising to “brighten” your complexion, which we all know is code for lighten it just a little. I think TikTok profits off people's insecurities.”

Meera's account reveals something Srnicek's (2017) platform capitalism framework describes structurally but cannot account for experientially: the specific feeling of watching as social space becomes a commercial one. Srnicek's (2017) framework establishes that TikTok extracts value from user participation yet does not explain how this extraction is felt as a betrayal, as the loss of something that once felt like conversation and now feels like a transaction. What Srnicek (2017) adds to her account is an explanation for why this shift is so effective. Platform capitalism, as he argues, does not merely provide a space in which commerce occurs; it engineers the conditions under which commerce becomes the path of least resistance. The commercial infrastructure of TikTok, shoppable links, in-app purchasing, seamless checkout, is designed to be frictionless, which means the transition from watching to buying happens before the user has formed a conscious intention to purchase. Meera knows this, which is why she can name it. But Naina's account shows what happens when that knowledge is not yet formed: she finds herself ‘halfway through a cart' without understanding quite how she arrived there. What Srnicek's (2017) framework reveals that Naina cannot see from within her own account is that this is not a lapse in judgement but the designed outcome of a platform that has deliberately collapsed the distance between aspiration and acquisition, because that collapse is where the revenue is.

**Sana:** “Conversations about skin tone on TikTok are like reality TV, loud, dramatic, and full of people pretending they're just educating while selling skin lightening solutions in the next breath. You've got creators live for 24 h straight, holding up jars of mystery cream like they've discovered the cure for melanin. Everyone's suddenly a dermatologist with a discount code.”

**Katrina:** “There's that Pakistani woman on TikTok who spends almost 12 h a day live-streaming from her basement, selling skin-lightening soaps and creams. Watching her promote those products nonstop made me realize how deeply embedded the idea is that lighter skin is more beautiful.”

Banet-Weiser (2018) argues that empowerment discourse is absorbed into commercial logic, but Sana captures the precise moment of that absorption, the point at which “just educating” becomes a cover for “selling a skin-lightening solution in the next breath”. Duffy (2017) provides a structural explanation for why the collapse is so complete. Her concept of aspirational labor describes how creators are structurally rewarded for performing authenticity while pursuing commercial ends; the incentive structure does not require deception; it simply makes authentic-feeling promotion more profitable than honest critique. TikTok's monetisation architecture, affiliate links, TikTok Shop integration, brand partnerships, gives creators a direct financial incentive to make commerce feel like community, and Duffy's (2017) logic explains why this is not a corruption of the platform's culture but an expression of its design. Katrina's account adds a dimension that Sana's does not make explicit: the intra-community dimension of this commerce. What her account reveals is the specific dissonance of watching a South Asian woman sell skin-lightening products to a South Asian audience, the way this transmits colourist ideology not as external imposition but as cultural self-reproduction. Drawing on Banet-Weiser's (2018) framework, peer credibility functions more effectively than institutional advertising precisely because it arrives as shared identity rather than sales. Content that appears to come from within the community carries a legitimacy that brand advertising cannot purchase, making it, structurally speaking, a more efficient vehicle for the same ideology.

### 4.3 Perceived algorithmic visibility and the reproduction of skin tone hierarchies on TikTok

Participants consistently described their experiences of TikTok as one in which lighter, filtered, or brightened faces dominate their For You Pages while darker or unfiltered appearances feel comparatively absent. While TikTok is often celebrated for its democratic potential, participants experienced it as producing an uneven terrain of visibility. These accounts speak to how the platform is perceived and felt, not to how its technical systems demonstrably function. TikTok's recommendation architecture is opaque to users, and this study makes no claims to verify algorithmic behavior from qualitative data. What the data reveal, and what constitutes this study's empirical claim, is that the perception of biased visibility is widespread, consistent, and consequential: it shapes self-image, engagement, and belonging.

**Ruksana:** “TikTok skincare trends make me feel like my natural skin tone is an unfinished project. According to the app, I'm apparently just a few serums, three brightening masks, and one miracle Korean essence away from finally being acceptable to the algorithm. Every time I scroll, I half-expect someone to announce the launch of a new improved human skin shade, now in TikTok beige!”

Ruksana's account reveals something that Noble's (2018) framework on algorithmic oppression cannot supply: the subjective phenomenology of being governed by an aesthetic standard you cannot see or name. Noble (2018) argues that algorithmic systems can encode racial bias; what Ruksana illustrates how the perception of that encoding is experienced as personal inadequacy. She does not say “the algorithm discriminates against my skin tone;” she says she is “just a few serums away from finally being acceptable.” The ideology has been successfully internalized as self-improvement. Ionescu and Licu (2023) then explain why internalization is so complete: TikTok's algorithm, as experienced by users, does not merely reflect preferences but actively shapes them, progressively narrowing the aesthetic window through which users understand themselves as acceptable.

**Kavita:** “To me, the real issue isn't that TikTok portrays beauty wrong, it's that it mirrors a society that still values fairness over authenticity. Changing TikTok means changing what we reward with our attention. Until people stop liking those videos, the algorithm will keep serving us the same lie.”

Kavita's account does something that platform critique alone cannot: it distributes responsibility. Noble (2018) and Benjamin (2019) locate the reproduction of racial hierarchy in algorithmic design; Kavita locates it in user behavior, “Until people stop liking those videos, the algorithm will keep serving us the same lie.” What her account reveals that theory could obscure is the complicity of the audience, including herself, in sustaining the system she critiques. This is not a finding that exonerates platform design; it complicates where the intervention needs to occur. Benjamin's (2019) concept of the New Jim Code, technological designs that encode racial preference while appearing neutral and responsive, captures precisely why individual refusal is insufficient. Love et al. (2025) show that skin-tone bias shapes click decisions within beauty content; users report being less likely to engage with videos featuring dark-skinned creators, and in systems that weight engagement signals, such disparities could contribute to reduced surfacing of such content, though the specific mechanics remain opaque to users. This supports Kavita's contention that user interactions sustain the repeated circulation of colourist content not through any single deliberate act but through the aggregated weight of many small choices, each of which appears innocent in isolation. Noble's (2018) framework, extended to engagement-optimized platforms, suggests that systems which learn from the full range of user behavior, including what users scroll past, how long they pause, and aggregated patterns across similar profiles, make individual acts of refusal insufficient to interrupt the system. The architecture learns from everything, which means the user can withdraw a single signal without withdrawing from the logic that produced it.

**Ayesha:** “TikTok skincare trends have made me more critical, not insecure, about my natural skin tone. It is basically colourism repackaged as self-care. What frustrates me most is how easily people fall for it. Creators push products that promise lighter skin, audiences buy into it, and the cycle keeps feeding itself. It's not just about skincare anymore, it's about selling insecurity.”

What Ayesha's account adds that Gill and Orgad (2021) cannot supply is the experiential texture of recognition from within: she has not merely observed the structure, she has felt it operate on her and chosen detachment as a response. This is a theoretically significant move. Gill and Orgad's (2021) critique of confidence culture describe how self-improvement is marketed as empowerment while quietly reinforcing dependence on consumer solutions; Ayesha's statement that “it's not just about skincare anymore, it's about selling insecurity” names this mechanism precisely. Her description of the self-perpetuating feedback loop, creators pushing products, audiences buying in, the cycle feeding itself, points to the commercial architecture Srnicek (2017) theorizes at the structural level. What Ayesha adds is the subjective corollary: wellness discourse places the imperative to improve inside the subject rather than outside it. It is not that society wants you to have lighter skin; it is that you want to glow, to radiate, to be your best self. The ideology is delivered through the notion of personal autonomy and arrives as self-determination. Ayesha's critical distance from this process does not place her outside it; it places her at its edge, able to name it while still inhabiting the platform it defines.

**Sabina**: “TikTok didn't just amplify the conversation about skin tone, it warped it. It turned something deeply personal into content, where people profit off the same insecurities they claim to challenge. On TikTok, it feels like they come from algorithms that know exactly what will make you doubt yourself.”

Sabina adds a dimension Ayesha does not name: the felt experience of being known by the system. Where Ayesha analyses the structure, Sabina describes the sensation, algorithms that “know exactly what will make you doubt yourself.” Ionescu and Licu (2023) reveal that this feeling of being known is not incidental to the platform's design but central to it: personalisation is the mechanism through which commercial platforms convert generalized trends into targeted purchase intent. The algorithm does not know what will make any individual user doubt herself, but it has learned, from aggregated behavioral data across users, which categories of content generate the kinds of engagement that precede purchasing decisions. The precision Sabina experiences is real. It is also the product of a system designed to produce exactly that feeling.

**Rekha**: “On TikTok, I've noticed that when South Asian or Sri Lankan creators with lighter skin post, the comments are full of compliments about their beauty. But when darker-skinned creators show up, the focus shifts, people start talking about their “features” or “attitude” instead, almost avoiding acknowledging their beauty directly. It's subtle, but it sends a clear message about which shades of brown are celebrated and which ones are tolerated.”

Rekha's account reveals something that Hall's (1990) theory of representation establishes at the level of structure: that within the category “South Asian,” a further hierarchy operates, distinguishing which “shades of brown are celebrated and which ones are tolerated.” Hall (1990) stipulates that representation reproduces power relations, but Rekha shows the precise texture of this reproduction in comment sections, in the shift from compliments about beauty to remarks about “features” or “attitude” as skin tone darkens. Theory describes the system; she documents its experience. Noble's (2018) analysis of Google Search provides a useful analogy here: algorithmic systems can learn from and amplify racially biased patterns of user engagement, producing discriminatory outputs through mechanics rather than deliberate design. Applied to TikTok's comment-driven visibility logic, the same pattern is plausible: the comments are not only expressions of individual prejudice but also inputs into a system that learns from them. The content that receives the most positive interaction shapes what the platform surfaces next, and what the platform surfaces next shapes what audiences learn to value. A similar recursive dynamic may be at work on TikTok, as Noble's (2018) framework suggests, though the specific mechanics remain opaque and this study makes no claim to verify them.

### 4.4 “It's just a preference”: colourism and the normalization of discrimination on TikTok

Hall (1990) describes the ideological work of representation as the process by which structural inequality is made to appear natural, individual, and unproblematic, a process in which “just a preference” becomes the discursive shield that protects colourist attitudes from critique. What Hall's (1990) framework cannot tell us is how this ideological work feels from the inside: whether it produces rage, exhaustion, resignation, or strategic detachment. The qualitative accounts in this theme reveal precisely this, showing that the normalization of colourist discourse on TikTok generates a range of affective responses that ideology critique alone cannot capture, and that these responses are shaped by the specific affordances of a platform where prejudice circulates at scale, anonymously, and without consequence.

**Meera:** “The truth is, TikTok didn't create these beauty standards; it just profits from them. People act shocked by how extreme some live sellers are, but they're only doing what gets attention and sales. Personally, I've learned to take it as a reminder of how powerful marketing and cultural conditioning can be. Instead of feeling pressured, it's made me double down on embracing my natural skin, because the moment you stop trying to fit their idea of beauty, you take your power back.”

Meera's account reveals that structural critique can function as an affective resource. Hall (1990) and Srnicek (2017) position TikTok as profiting from pre-existing beauty hierarchies, but they cannot tell us that naming this mechanism might, for some users, convert potential shame into defiance. Meera has arrived at something resembling what Freire (1970) terms critical consciousness, the capacity to perceive and name the structural forces shaping one's experience, allowing her to observe the system from within it and refuse its terms. Banet-Weiser (2018) would recognize this as the absorption of resistance into commodity form, not to negate Meera's agency, but to situate it within the structural conditions that simultaneously enable and contain it. What Meera's account adds that neither Freire nor Banet-Weiser can supply is the specific affective texture of this position: the feeling of clarity without exit. She can name the system, refuse its terms, and remain inside it simultaneously, not because her refusal is incomplete but because the platform offers no outside from which to refuse. That is not a failure of critical consciousness; it is its limit condition under platform capitalism.

**Sana:** “So many videos promote brightening or lightening products, and even if it's subtle, it sends a message that lighter or more even-toned skin is somehow better. What makes it worse is the comment sections. I've noticed how people with darker or more textured skin often get targeted with racist or Islamophobic remarks, especially lately in the UK. It's exhausting trying to unlearn that pressure, but I've realized that no amount of product can change how society views skin. It's the mindset that needs to change, not the melanin.”

Sana's account reveals the intersection of colourist beauty discourse with Islamophobia as a distinct and compounding form of racialised hostility. Tate (2018) and Glenn (2008) theorize colourism as a system organized around skin tone; Sana's experience indicates that on TikTok, this system is not separable from religious identity; darker skin and visible Muslim identity are targeted together in the comment sections she describes, producing a layered vulnerability that skin-tone frameworks alone cannot capture. Banet-Weiser's (2018) argument about popular misogyny, the coexistence of celebratory and harmful cultural narratives within the same digital space, explains why hostile and prejudiced responses in comment sections are not incidental failures of moderation but structural features of a platform whose engagement-reward mechanisms, as participant experienced it, create conditions in which hostile responses circulate without consequence.

The platform does not distinguish between the affect of admiration and the effect of contempt; both keep users scrolling, both generate the metric that matters. Her conclusion that “it's the mindset that needs to change, not the melanin” locates the conflict at the level of ideology, but Srnicek (2017) adds a structural account of why mindsets alone are insufficient: they are produced, sustained, and amplified by platform architectures whose commercial logic gives them no incentive to change.

**Deepa:** “I feel like conversations on TikTok about skin tone mimic the conversations in real life but are used with sort of politically correct terms. Those on TikTok do it in a sly way, they won't outwardly tell me that being dark is ugly the way my family does, but they'll say, “brighten that skin for luscious skin” as if my skin was already an issue.”

Deepa makes a distinction that is theoretically crucial and empirically precise: she identifies TikTok not as a space where colourism is more intense than in offline life, but as a space where it is more linguistically sophisticated, positioned through the messaging of wellness and self-care. This is exactly what Parameswaran and Cardoza (2009) describe in their analysis of fairness discourse in South Asian beauty advertising: the substitution of explicit racial hierarchy with the coded language of improvement, a substitution that is commercially rational as well as ideologically effective because wellness language is more searchable, more shareable, and less likely to generate content moderation flags than explicit colourism. Srnicek (2017) makes the structural logic of this explicit: platform capitalism shapes the form content takes as much as its volume, rewarding those whose language aligns with its optimisation logic regardless of whether creators are conscious of that alignment. Deepa contributes the experiential texture of this substitution, the specific feeling of having one's skin described as an “issue” through language that presents itself as helpful. The platform does not require creators to be conscious of this optimisation: it simply rewards those whose language aligns with it.

**Zulaykha:** “Conversations about skin tone on TikTok feel very different from the ones I have in my everyday life. With my friends and family, there's an understanding. But on TikTok, those conversations can feel a lot harsher and more exposed. While the platform allows people to speak openly about racism and discrimination, it also gives space for racist and Islamophobic voices to spread hate without consequence. It makes me more cautious about what I share and sometimes even afraid to show my face online. In real life, there's compassion and understanding, but on TikTok, it often feels like you're constantly defending your right to exist in your own skin.”

Zulaykha's account reveals a distinction between being seen and being exposed. Visibility on TikTok, as she experiences it, does not produce the recognition and belonging that Sujon and Ntalla (2025) identify as its potential reward. Instead, it produces hypervisibility, a condition in which one's presence invites not celebration but scrutiny, hostility, and the demand to justify one's right to participate. This extends what Sujon and Ntalla (2025) began to theorize: that for racialised women, digital visibility can function as a form of vulnerability rather than empowerment, and that this vulnerability is relational, shaped by the contrast with the safety of offline community spaces where, as Zulaykha describes, “there's compassion and understanding”. Sujon and Ntalla (2025) argument positions visibility on TikTok as simultaneously a symbolic reward and a source of precarity for marginalized creators, but Zulaykha's account specifies the relational mechanism that makes this precarity felt: it is the contrast with the accountability of offline community that makes the platform's exposure so acute. Srnicek's (2017) platform capitalism adds a structural explanation for why this contrast exists: offline community norms are maintained through social accountability between known individuals; TikTok's scale-amplified environment provides no equivalent mechanism, and its commercial logic gives it no incentive to create one. Hate comments can circulate “without consequence” not as a failure of individual conduct but as a structural property of a platform designed to maximize engagement without acknowledging what that engagement costs those it targets.

### 4.5 Colourist shame on TikTok: transmission and resistance

Tate's (2018) concept of the governmentality of beauty shame holds that colourism operates not through explicit command but through the internalization of norms that subjects apply to themselves, a self-surveillance in which shame is both the mechanism of control and its primary affective output. What the concept cannot supply is an account of how this self-surveillance is triggered, sustained, and occasionally broken in a specific digital environment: the precise mechanisms through which a TikTok scroll converts into felt bodily inadequacy. The narratives below make that process visible, showing that TikTok does not simply reflect colourist norms but provides the specific affordances, peer-to-peer transmission, algorithmic repetition, seamless commerce, through which those norms are renewed as personal feeling.

**Meera:** “My friends will swear they're just trying to even out their skin tone while holding a product literally called Whitening Miracle. TikTok is the loud, global version of what our communities already whisper, just with filters, hashtags, and a checkout link.”

Meera articulates what platform capitalism theory (Srnicek, 2017) describes structurally but cannot account for experientially: the continuity between offline community norms and their digital amplification. Srnicek (2017) can tell us that TikTok commodifies user participation and scales cultural content for profit; he cannot tell us that the specific content being scaled is the same colourist ideology that participants encounter in their kitchens and living rooms. What Meera's account adds to theory is the lived experience of scale, TikTok does not introduce colourism to her social world, it turns up its volume to a global frequency, attaching a checkout link to what was previously a whispered family pressure. Duffy's (2017) account of aspirational labor shows how affect, including shame, aspiration, and the desire to improve, is not merely a byproduct of platform participation but the primary resource platform capitalism extracts and monetises. The whispers become a checkout link not by accident but because shame is commercially productive: it generates the emotional state that precedes the purchase decision. The Whitening Miracle is not incidentally named; the promise encoded in that name is precisely what generates the click, the sale, and the platform's revenue. Platform capitalism, as Srnicek (2017) theorizes it, engineers the conditions under which commerce becomes the path of least resistance; the specific vehicle here, seamless purchasing, personalized targeting, frictionless checkout, is designed to convert the emotional state shame produces into revenue before it dissipates.

**Leela:** “I have felt pressured to use skincare products because of the way skin tones are portrayed on TikTok. As a dark-skinned Sri Lankan, I naturally have melasma and hyperpigmentation, it had never bothered me so much until I started seeing other women point it out on TikTok to get rid of because it's patchy or uneven, and my natural ethnic complexion is supposed to be this unattainable white board. Due to how normalized these trends became I did invest in some skin care products for whitening, but with support I eventually understood the toxicity of it all.”

Leela's account is the dataset's most precise illustration of how Tate's (2018) governmentality of beauty shame operates at the level of the individual body. Tate (2018) argues that colourism functions through internalized self-regulation; the subject applies the dominant aesthetic standard to herself, producing shame not as an external imposition but as a felt conviction about one's own inadequacy. What Leela's narrative contributes that the theoretical framework cannot predict is the specific trigger: it was not advertising, family pressure, or cultural tradition that made her melasma and hyperpigmentation feel like problems, but the cumulative encounter with other women on TikTok pointing out these features as defects. This peer-to-peer mechanism, shame transmitted through participatory content rather than top-down broadcast, is a distinctly digital form of the governmentality Tate (2018) describes, and one that her framework, developed prior to the TikTok shop marketplace, does not fully anticipate. Leela's own trajectory offers a structural account of why this process required external support to undo: the platform's design ensures continuous re-exposure to the norms that produced the shame, making individual critical reflection insufficient. Tate (2022) describes decolonial self-articulation as a social and relational process; Leela's account confirms this precisely, showing that the exit from internalized shame was not achieved alone.

**Katrina:** “The platform claims to celebrate diversity, but the view of beauty that dominates my feed still feels pretty constrained. Light, clear, glowing skin is usually what gets pushed to the top. That said, I do see some dark-skinned girlies showing up on my For You Page, and I love that. They're not the majority, but the handful who do appear really challenge those narrow ideals and make me feel seen. At this point, I'm convinced TikTok doesn't sell skincare, it sells shame in tiny glass bottles.”

Katrina's account holds two emotions simultaneously without resolving them, a complexity that shame theory and platform critique alone cannot account for. She experiences TikTok as a space of constrained beauty ideals and as a space where she occasionally feels genuinely seen, and she does not reconcile these two experiences into a single verdict. This ambivalence is analytically significant because it resists the totalising logic of both critical platform studies (van Dijck et al., 2018; Gillespie, 2018), which tend to emphasize harm, and empowerment frameworks (Banet-Weiser, 2018; Gill and Orgad, 2021), which tend to emphasize agency. What her data reveals is that the same platform, and sometimes the same feed, can function as both a site of shame and a site of recognition depending on what surfaces. Harriger's (2023) content analysis, showing that over 93% of body-positivity content reflected Western beauty ideals, combined with the audience engagement patterns identified by Love et al. (2025), suggests that the moments of visibility Katrina describes as exceptional are structurally produced as exceptional: they are ruptures in a pattern rather than evidence that the pattern is changing.

**Rekha:** “When I saw that TikTok video of Chunkz saying, “Ehhh, Sri Lanks come like that,” my first reaction was honestly, what the hell do you mean by that? It wasn't just a random comment. It reflected a deeper perception that Sri Lankans, and South Asians with darker skin in general, can't be considered pretty. Hearing it said so casually, in a way that got laughs online, really highlighted how normalized colourism still is, even among people who claim to celebrate diversity.”

Rekha's reaction to a viral joke identifies a mechanism that neither Tate's (2018) shame framework nor Hall's (1990) representation theory fully accounts for: the role of humor as a vehicle for colourist normalization within intra-community spaces. Tate (2018) theorizes shame as something imposed by dominant beauty standards on racialised subjects; Hall (1990) theorizes representation as a site where ideological meanings are produced and contested. Neither fully anticipates the situation Rekha describes in which a South Asian influencer performs colourism as comedy to an audience that rewards it with laughter. What her account reveals is the affective precision of this mechanism: the joke works, and causes harm, precisely because it comes from within the community. It carries the implicit authority of shared cultural knowledge while simultaneously weaponising that knowledge against some members of the community. Banet-Weiser's (2018) concept of popular misogyny, the coexistence of celebratory and harmful cultural narratives within the same space, applies here as popular colourism. The platform's engagement logic does not distinguish between the laughter of solidarity and the laughter of contempt. Both generate the same metric.

Alongside these narratives of internalization and shame, Priya articulates explicit resistance:

**Priya:** “Do these trends affect how I feel about my skin? Absolutely. They make me want to delete the app, pour my moisturizer down the drain, and start a countertrend called #UnevenAndUnbothered. Because if TikTok's version of “perfect” skin is the standard, then honestly, I'd rather fail the test.”

Priya's response reveals what Tate's (2018) framework on beauty shame implies: that the refusal of the dominant aesthetic standard is itself an affective achievement, not simply an intellectual position. She does not merely disagree with TikTok's beauty norms; she describes a visceral, embodied response, “delete the app, pour my moisturizer down the drain,” that signals how deeply the standard has registered even in the act of rejecting it. What her account adds to Tate's (2018) framework is the phenomenology of resistance: the feeling of what it is like to refuse, from the inside, a norm that has enough gravitational pull to produce that strength of reaction, a dimension Tate (2018) theorizes at the structural level but does not supply at the experiential one. Sujon and Ntalla (2025) add a structural qualification Priya does not make: visibility on TikTok functions as both reward and constraint, meaning that her proposed countertrend #UnevenAndUnbothered would, if it gained traction, generate the same engagement that sustains the platform whose logic she is critiquing. Banet-Weiser (2018) extends this further: resistance is frequently absorbed as a legible and marketable form of authenticity, circulated by the same commercial infrastructure it sets out to refuse. Priya's refusal is real and meaningful. It is also, structurally speaking, a content format.

## 5 Discussion and concluding remarks

This study investigated how British South Asian women perceive, interpret, and navigate brightening-related skincare content on TikTok, with particular attention to how colourist beauty norms are reproduced, rationalized, and, at moments, contested within a platform environment shaped by commercial and algorithmic logics. The discussion proceeds in three stages. It first provides a direct answer to each of the study's three research objectives, identifying not only what the data show but what they reveal that the existing literature had not yet established. It then theorizes the central analytical finding, the tension between internalization and resistance, as a structural property of platform capitalism rather than a feature of individual psychology. Finally, it sets out the study's contributions and their implications for colourism research, feminist digital media studies, and platform scholarship.

### 5.1 What the study found and what it adds

The first objective asked how engagement with TikTok skincare content shapes self-perceptions and aspirational beauty ideals among British South Asian women. The study's answer is this: colourist norms are transmitted primarily through aesthetic repetition and linguistic repackaging rather than through explicit racial instruction. Participants were not told that lighter skin is more desirable; they encountered this message through the For You Page, in the vocabulary of brightening, glow and radiance, in the visual dominance of smoothed and lightened complexions, and in the implicit logic of what counts as skin that is “fixed” vs. skin that is really good enough.

This is not a finding the existing literature had established. Glenn (2008) and Parameswaran and Cardoza (2009) established that skin-lightening discourse is coded through euphemistic wellness language in South Asian beauty advertising. What this study adds is evidence of how that coding operates in a participatory, peer-mediated, algorithmically amplified environment where the source of the message is not a brand but an ordinary user, often a South Asian woman herself, whose apparent relatability makes the colourist logic she transmits harder to identify and easier to internalize. Kiran's observation that TikTok reproduces ‘the same old colourism, just with trendier language' names this mechanism precisely; what the study contributes theoretically is an account of why the new packaging is more effective than the old one: because it arrives through a face that looks like yours, on a platform that feels like community.

The second objective asked how British South Asian women experience and interpret color-related terminology in TikTok skincare content, and how this shapes their perceptions of ideal skin tone. The study's answer is that the relationship between exposure and internalization is real but not uniform, and that critical awareness mediates but does not neutralize affective impact. This finding departs meaningfully from the existing literature in two directions. First, it challenges frameworks that treat exposure to colourist content as straightforwardly damaging, social comparison theory (Festinger, 1954; Vogel et al., 2014) and body dissatisfaction research (Schröder and Behm-Morawitz, 2025) tend to model exposure as producing a predictable negative outcome. The data here show a more complex picture: participants who demonstrated structural awareness of colourist discourse, who could name platform capitalism, trace colonial histories, and critique influencer rhetoric, still reported affective vulnerability. Ruksana's simultaneous “admiration and frustration,” Ayesha's critical detachment coexisting with consumer temptation, and Kavita's structural analysis of the algorithm alongside her continued scrolling all indicate that critical literacy is a protective resource but not a shield. Second, the study finds that the modulating factor is not simply knowledge but the relational and cultural context in which users are embedded, what participants have been told about their skin by family, what community narratives they carry, and whether they have access to counter-discourses that affirm darker complexions. This relational dimension of internalization is undertheorised in the existing digital media literature, which tends to focus on platform exposure in isolation.

The third objective asked whether and how TikTok's structures, as perceived by users, commodify colourist skincare narratives and influence consumption. The study's answer is unambiguous: they do, and the mechanism is more sophisticated than prior accounts of influencer marketing suggest. Existing scholarship on platform capitalism (Srnicek, 2017) and aspirational labor (Duffy, 2017) establishes that digital platforms extract value from user participation and that creators commodify authenticity for commercial gain. What this study adds is an account of how this commodification operates at the level of the audience, not only the creator. Naina's account of finding herself mid-way through a cart of brightening serums without having consciously decided to buy them illustrates a process Srnicek and Duffy do not fully theorize: the conversion of ambient algorithmic exposure into purchasing behavior through the accumulated affective weight of repeated aesthetic encounters, without the consumer explicitly endorsing the ideology that drives the purchase. The commercial architecture of TikTok, seamless in-app purchasing, shoppable livestreams, the absence of clear distinctions between organic and sponsored content, creates conditions in which colourist desire does not need to be consciously held to be commercially productive. It need only be felt, briefly, in the moment before the checkout link is tapped.

This study makes three contributions to the literature. First, it advances the theorisation of colourism in digital spaces by showing that the platform reproduction of colourist norms operates not only through content, the presence of brightening products, lighter-skinned creators, and Eurocentric beauty standards, but through the affective and commercial architecture of the platform itself. Existing scholarship has established that algorithmic systems can encode racial bias (Noble, 2018; Benjamin, 2019) and that influencer culture commodifies racialised femininity (Duffy, 2017; Taylor, 2025). What this study adds is an account of the user-side experience of these processes: how colourist norms come to feel like personal preference, community wisdom, and authentic self-care rather than structural imposition. This distinction matters because it explains why colourist ideology is so resistant to critique even among participants who can identify it, the ideology does not present itself as ideology.

### 5.2 The internalization-resistance tension: a structural account

The most significant finding across all five themes is not any single instance of colourist harm or individual act of resistance, but the consistent coexistence of both within the same participants, the same accounts, and sometimes the same sentences. This is the study's central analytical contribution, and it requires a structural rather than psychological explanation, one that accounts for why this tension is so stable and so widespread rather than treating it as a feature of particular individuals' ambivalence.

Tate's (2018) concept of the governmentality of beauty shame provides the theoretical foundation. For Tate, colourism does not operate primarily through external command, through being explicitly told that darker skin is inferior, but through the internalization of regulatory norms that subjects apply to themselves. Shame is the affective mechanism through which this self-regulation is maintained: it motivates self-surveillance, self-correction, and the consumption of products that promise to bring the body into conformity with the dominant aesthetic standard. What TikTok adds to this framework, and what this study documents, is a set of platform-specific mechanisms through which the governmentality of beauty shame is continuously renewed. The For You Page, as participants consistently describe it, means that the aesthetic standard is not encountered once but repeatedly, in content that feels spontaneous and authentic rather than prescriptive. The peer-to-peer transmission of colourist norms, through ordinary users pointing out each other's melasma, through influencers framing skin-lightening as self-care, through comment sections rewarding lighter-skinned creators with compliments and darker-skinned creators with remarks about their “features,” means that the source of shame is diffuse and relational rather than institutional and identifiable. This is precisely what makes it so difficult to refuse: there is no single authority to reject, no advertisement to dismiss, only the accumulated weight of what appears to be community consensus.

And yet participants do refuse, partially, provisionally, and often simultaneously with complying. This is the finding that Tate's (2018) framework illuminates but does not fully resolve. Tate (2022) theorizes decolonial self-articulation as the process through which racialised women come to name, refuse, and reframe dominant beauty ideals. Leela's trajectory, from never having found her hyperpigmentation troubling, to purchasing whitening products, to “understanding the toxicity of it all” with external support, follows this arc. Meera's strategic detachment, Priya's embodied refusal, Ayesha's critical distance: all represent forms of decolonial self-articulation in practice. But what Tate's framework does not account for is the structural condition that this study foregrounds: the platform does not distinguish between compliant and resistant content. Both generate engagement. Both feed the same commercial system. This is where Banet-Weiser (2018) becomes indispensable. Her concept of popular feminism, the absorption of feminist and empowerment discourse into commercial and media logics, applies here as popular decolonialism: the language of skin-tone pride, of #BrownSkinGirl and #DarkIsBeautiful, of refusing brightening culture, circulates on TikTok as content, generating the views and engagement that sustain the platform economy that sustains the colourist beauty industry. Resistance is not co-opted after the fact; it is structurally incorporated from the beginning, because the platform's engagement logic makes no distinction between the affect of shame and the affect of defiance. Both keep users scrolling.

This structural incorporation of resistance is what makes the internalization-resistance tension not a psychological paradox but a political one. The question it poses is not why individual women feel both pressured and defiant, that is entirely understandable, but why the system is designed such that both responses are commercially equivalent. The answer this study points toward is that platform capitalism, as Srnicek (2017) describes it, is indifferent to the content of affect; it requires only that affect be sustained and that it drives engagement. Colourist shame and anti-colourist pride are, from the platform's perspective, the same thing: content. This is the structural insight that neither Tate's framework nor Banet-Weiser's alone fully captures, and that this study's data make visible, not through individual testimony but through the pattern that emerges when individual testimonies are read together. The tension between internalization and resistance is not a personal failing or an incomplete politics; it is the expected outcome of a system designed to monetise both.

This has a specific implication for how we theorize agency in digital environments. Existing frameworks in feminist digital media studies, including algorithmic oppression (Noble, 2018), surveillance capitalism (Zuboff, 2019), and popular feminism (Banet-Weiser, 2018), read together, can be seen to oscillate between two positions: one that emphasizes structural constraint and renders users as relatively passive recipients of platform ideology (Noble, 2018; Zuboff, 2019; Elias and Gill, 2018), and one that emphasizes user agency and renders resistance as inherently meaningful and politically generative (Banet-Weiser, 2018; Jackson et al., 2020). Both positions, the data suggest, are partially correct and structurally insufficient. What is needed, and what this study begins to develop, is a framework that holds structural constraint and meaningful agency together without resolving the tension between them, because that tension is not a theoretical problem to be solved but an empirical condition to be theorized. British South Asian women on TikTok are not dupes of colourist ideology, nor are they free agents who can simply choose to refuse it. They are subjects navigating a system that has been designed, at every level, to make both compliance and resistance feel like personal choices and to profit from both.

### 5.3 Contributions and limitations

The preceding analysis points toward three contributions that extend beyond the specific findings and carry implications for how colourism, platform scholarship, and feminist digital media studies are theorized. First, the study advances understanding of how colourist norms are reproduced in digital spaces, not only through the visible presence of brightening products, lighter-skinned creators, and Eurocentric beauty standards, but through the affective and commercial architecture of the platform itself. Where prior scholarship has documented structural bias at the level of algorithmic design and influencer economy, this study accounts for what that bias feels like from the inside: how colourist norms come to feel like personal preference, community wisdom, and authentic self-care rather than structural imposition. This matters because it explains why colourist ideology is so resistant to critique even among participants who can identify it; the ideology does not present itself as ideology, and any intervention that addresses only its visible content will leave its affective infrastructure intact.

Second, the study contributes to feminist and diasporic media research by establishing that British South Asian women on TikTok are not simply recipients of colourist discourse but active theorists of it. Participants in this study consistently demonstrated a form of critical media literacy, the capacity to name platform capitalism, trace colonial genealogies of skin-tone hierarchy, and identify the commercial mechanisms through which colourist desire is produced, that has not been foregrounded in prior research on this community's digital media engagement. This critical literacy is not straightforwardly protective, as the data show; but its existence is analytically and politically significant. It indicates that the resources for resistance are already present within the community, and that the obstacle to more sustained refusal is not ignorance but structural: the platform is designed to absorb critique as content.

Third, by centring the internalization-resistance tension as its primary analytical object rather than treating it as a complication of a simpler finding, this study makes a methodological argument for qualitative research in this area. Only an approach capable of holding contradiction, of sitting with ambivalence rather than resolving it into a cleaner narrative, can capture what is actually happening when British South Asian women engage with TikTok's beauty culture. The study does not conclude that TikTok is harmful or that resistance is futile; it concludes that both harm and resistance are structurally produced by the same system, and that understanding that system requires methods that can follow participants into the complexity of their own experience.

For British South Asian women, navigating TikTok involves a negotiation that is never finally resolved, because the platform ensures that every act of participation, including refusal, feeds the same commercial infrastructure. This is not a counsel of despair. It is a precise diagnosis of where the structural problem lies not in individual users' responses to colourist content, but in the design of a system that makes colourist norms profitable and embeds them in the everyday rhythms of digital leisure. Addressing that problem requires intervention at the level of platform design, content moderation policy, and advertising regulation, not at the level of individual media literacy.

## 6 Limitations and future research

Although this study offers valuable qualitative insights into the intersections of TikTok, colourism, and self-perception among British South Asian women, several limitations must be acknowledged. The sample of 15 participants is appropriate for a qualitative study of this kind, where analytical depth and contextual richness are the goal rather than statistical representation. Generalisability is not claimed. In addition, the use of snowball sampling with existing British South Asian digital and community networks means participants may share overlapping social circles, cultural reference points, or attitudes toward colourism and beauty norms, which could limit the diversity of perspectives captured to a more dispersed sampling strategy. This is a recognized limitation of snowball sampling more broadly, and future research could mitigate this by combining snowball recruitment with other strategies, such as purposive sampling across more varied networks, to capture a wider range of viewpoints. The descriptive survey items included in the data collection were used solely as contextual framing and carry no inferential weight; future research seeking to examine the scale or distribution of these patterns across populations would require a larger, purposively sampled study with a properly powered quantitative design. Future qualitative research could also expand the participant pool and explore similar themes across other cultural and diasporic groups to understand how colourism and algorithmic visibility manifest differently across communities. Including participants with diverse identities spanning gender, sexuality, ability, and age would further enrich understanding of how intersecting experiences shape digital beauty perceptions.

A further limitation lies in the absence of a content analysis of TikTok videos. This study focused primarily on participants lived experiences and perceptions; however, analyzing captions, hashtags, audio, and visuals could provide a more comprehensive picture of how colourist language and aesthetics circulate on the platform, and would allow future researchers to examine the gap between user perception of algorithmic behavior and what content analysis can observe. Incorporating content analysis in future research would help capture how TikTok's short-form design and algorithmic logic contribute to the visibility and virality of colourist narratives.

Finally, future studies might explore the relationship between digital self-presentation and body image more explicitly. Burnell et al. (2022) found that frequent photo retaking and filter use correlated with body image concerns, suggesting that self-curation itself may heighten dissatisfaction. Building on these insights, future research should examine how TikTok's culture of filters, virality, and algorithmic validation influences users' emotional wellbeing and reinforces or disrupts colourist ideals, ideally drawing on longitudinal designs that can capture change over time.

## Data Availability

The raw data supporting the conclusions of this article will be made available by the authors, without undue reservation.
